# Supplementary material for: Structures of the human spliceosomes before and after release of the ligated exon
Source: Cell Res. 2019 Feb 6;29(4):274–85. doi: 10.1038/s41422-019-0143-x (PMC6461851; doi:10.1038/s41422-019-0143-x)
Supplement: Supplementary file 1 — Supplementary Figure 1 [file 41422_2019_143_MOESM1_ESM.pdf]

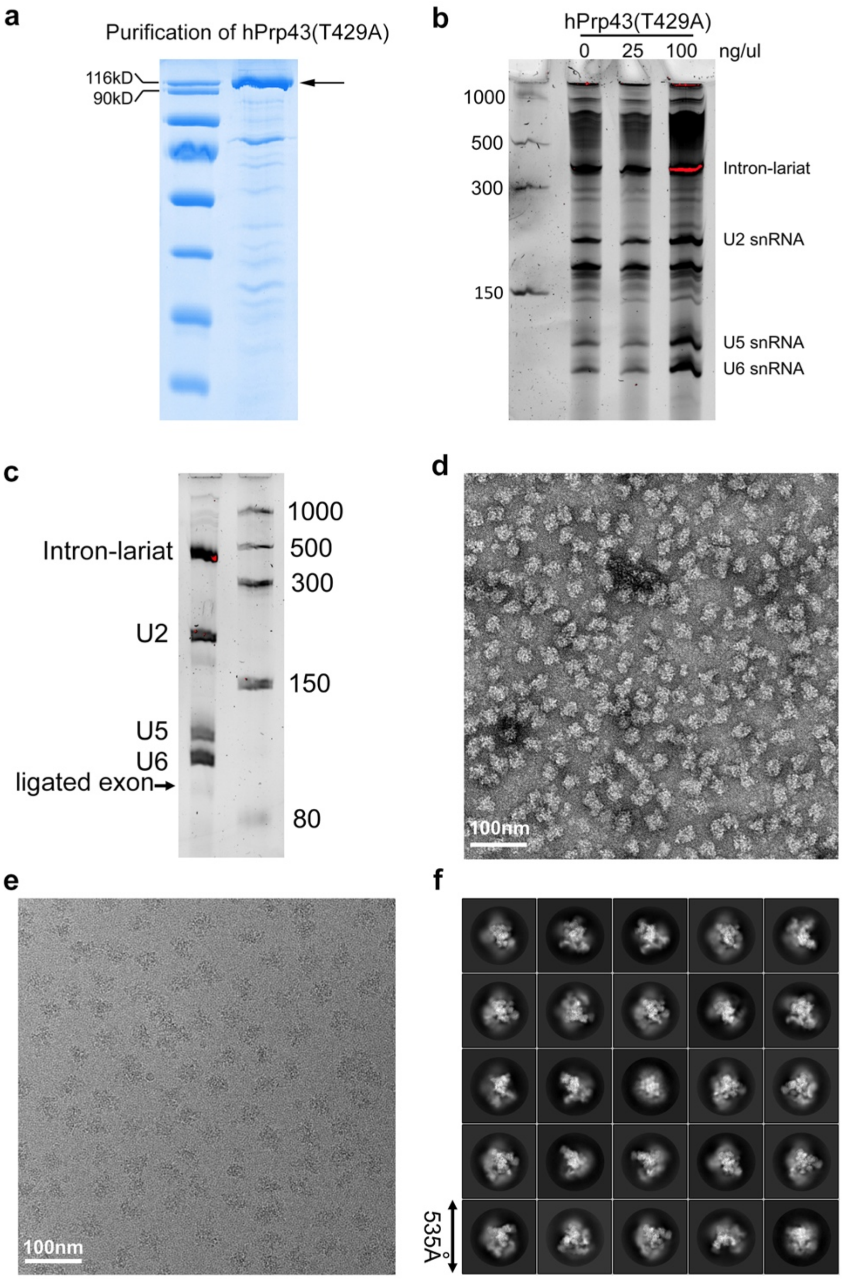

Supplementary information Figure S1. Purification and EM analysis of the human spliceosomal P and ILS complexes. a, The purified mutant protein Prp43 (T429A) is visualized by SDS-PAGE and Coomassie staining. **b,** Representative results of an intron release experiment shown on an RNA-urea gel. The splicing reaction was performed in the presence of varying amounts of hPrp43(T429A). The RNA elements of the ILS complex are indicated. **c,** An RNA gel showing the RNA components of the spliceosomal complexes after MS2-MBP affinity purification and glycerol gradient centrifugation. **d,** A representative electron microscopy (EM) micrograph of the final sample stained by uranyl acetate. Scale bar: 100 nm. **e,** A representative cryo-EM micrograph of the final sample. Scale bar: 100 nm. **f,** Representative 2D class averages calculated for the P and ILS complexes.
