# Supplementary material for: Structures of the human spliceosomes before and after release of the ligated exon
Source: Cell Res. 2019 Feb 6;29(4):274–85. doi: 10.1038/s41422-019-0143-x (PMC6461851; doi:10.1038/s41422-019-0143-x)
Supplement: Supplementary file 2 — Supplementary Figure 2 [file 41422_2019_143_MOESM2_ESM.pdf]

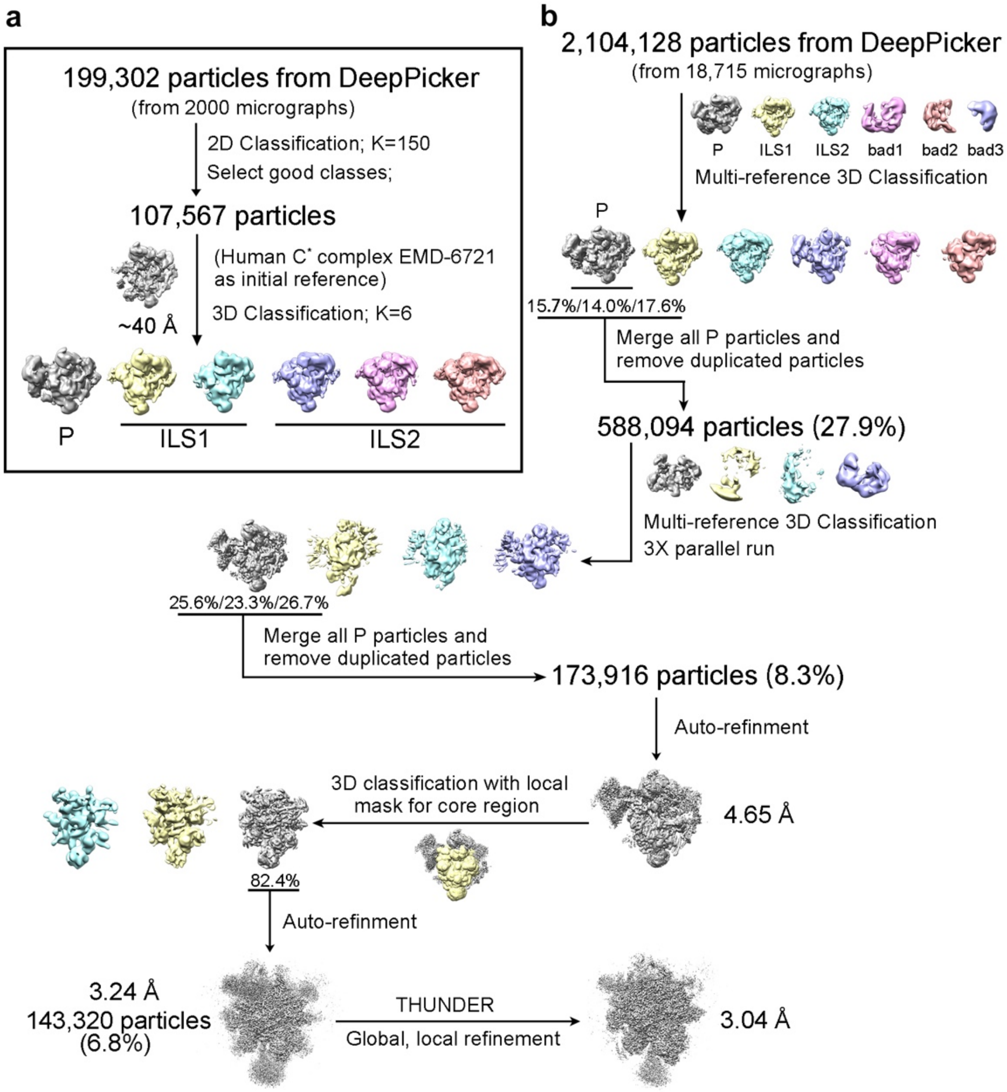

Supplementary information Figure S2. Preliminary cryo-EM data processing and structure determination of the human spliceosomal P complex. **a**, Analysis of the initial dataset of 2,000 micrographs of the affinity-purified human spliceosomal complexes. This analysis reveals that the vast majority of the particles belong to either the P complex (with ligated exon) or the ILS complex (ligated exon already dissociated). The ILS complex has two distinct conformations: one with Prp43 (ILS2) and one without Prp43 (ILS1). **b,** A flow chart description of the EM data processing and structure determination for the human spliceosomal P complex. The final reconstruction has an average resolution of 3.04 Å. Please refer to Methods for details.
