# Supplementary material for: Structures of the human spliceosomes before and after release of the ligated exon
Source: Cell Res. 2019 Feb 6;29(4):274–85. doi: 10.1038/s41422-019-0143-x (PMC6461851; doi:10.1038/s41422-019-0143-x)
Supplement: Supplementary file 3 — Supplementary Figure 3 [file 41422_2019_143_MOESM3_ESM.pdf]

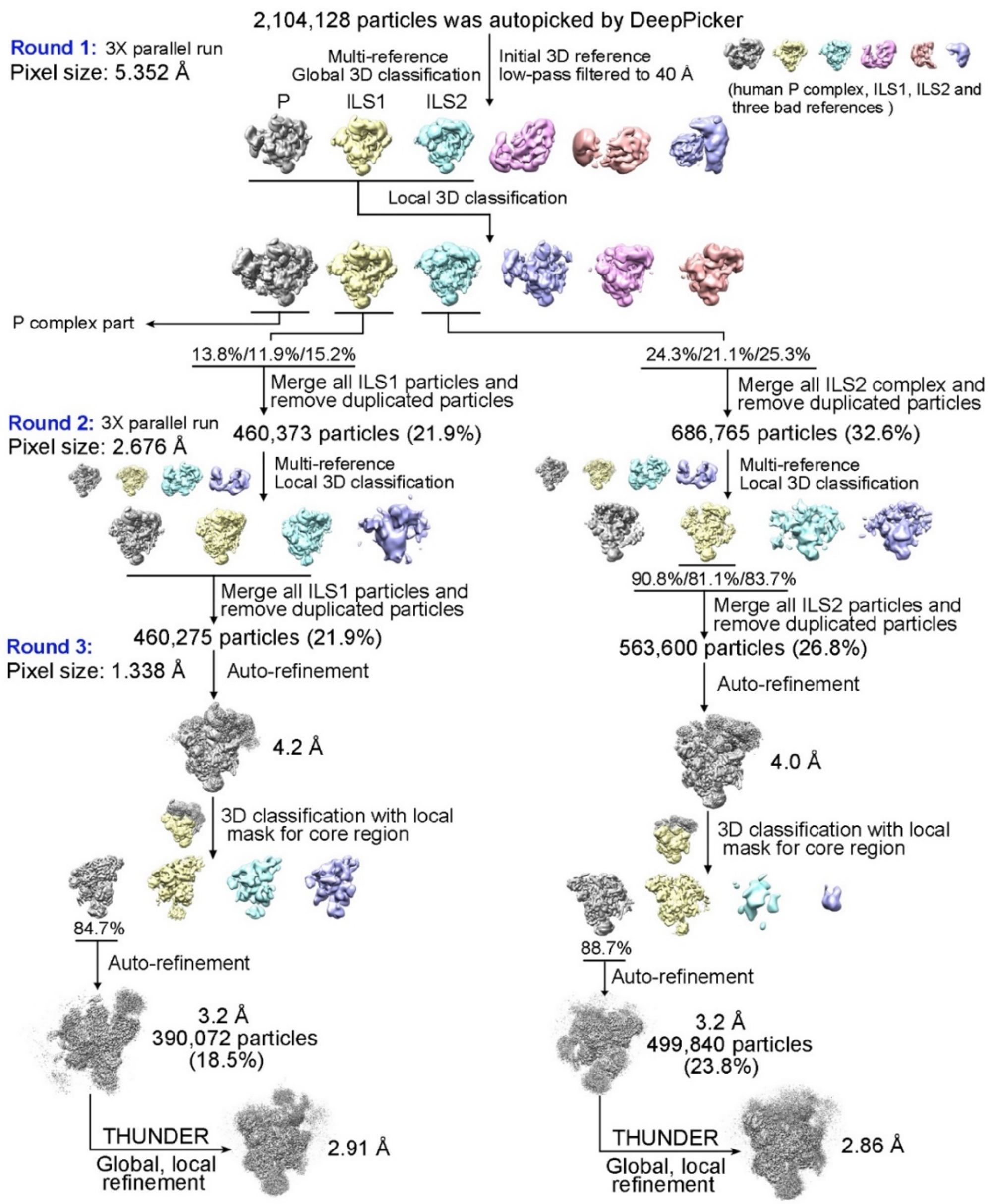

Supplementary information Figure S3. A flow chart description of the EM data processing and structure determination of the human spliceosomal ILS complexes. On the basis of the FSC value of 0.143, the final reconstruction has an average resolution of 2.91 Å for the ILS1 complex (prior to Prp43 recruitment) and 2.86 Å for the ILS2 complex (after Prp43 recruitment). Please refer to Methods for details.
