# Supplementary material for: Structures of the human spliceosomes before and after release of the ligated exon
Source: Cell Res. 2019 Feb 6;29(4):274–85. doi: 10.1038/s41422-019-0143-x (PMC6461851; doi:10.1038/s41422-019-0143-x)
Supplement: Supplementary file 4 — Supplementary Figure 4 [file 41422_2019_143_MOESM4_ESM.pdf]

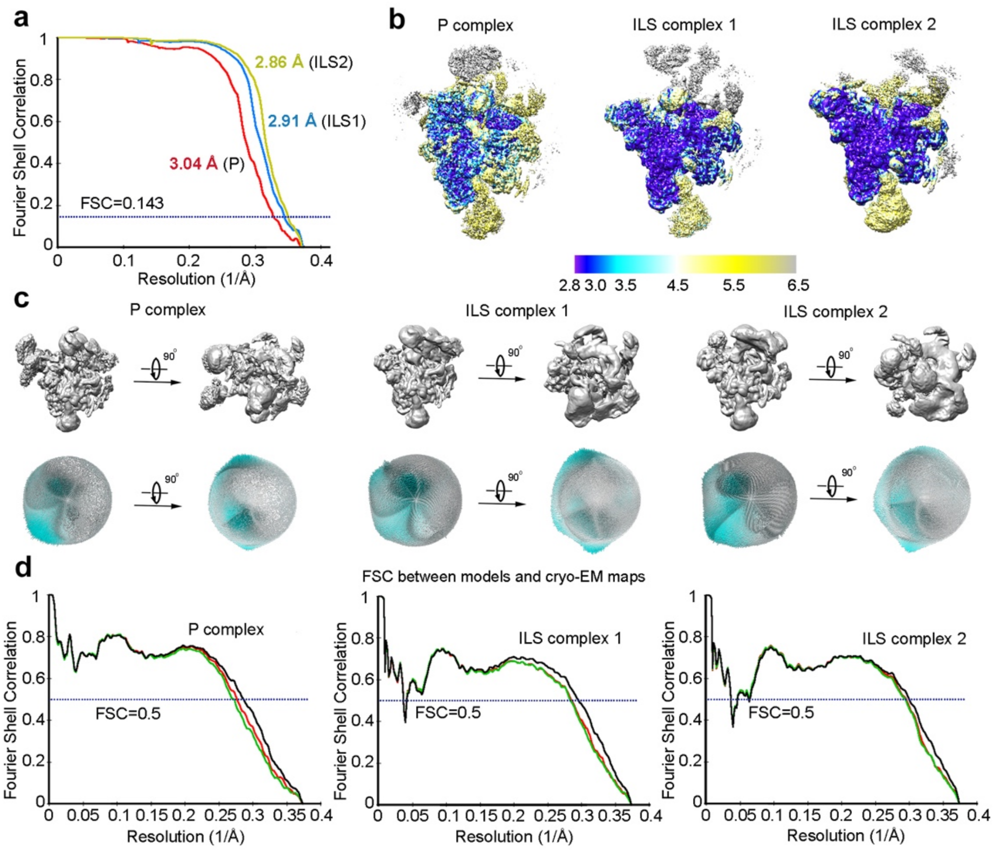

Supplementary information Figure S4. Cryo-EM analysis of the human spliceosomal P and ILS complexes. a, The average resolution is estimated to be 3.04 Å, 2.91 Å and 2.86 Å for the overall reconstruction of the P, ILS1, and ILS2 complexes, respectively, on the basis of the FSC criterion of 0.143. **b,** The local resolutions are color-coded for different regions of the P, ILS1, and ILS2 complexes. **c,** Angular distribution of the particles used for the reconstruction of the spliceosomal P, ILS1, and ILS2 complexes. Each cylinder represents one view and the height of the cylinder is proportional to the number of particles for that view. **d,** The FSC curves of the final refined model versus the overall map it was refined against (black); of the model refined in the first of the two independent maps used for the FSC calculation versus that same map (red); and of the model refined in the first of the two independent maps versus the second independent map (green). The generally similar appearances between the red and green curves indicates that the refinement of the atomic coordinates did not suffer from severe over-fitting.
