# Supplementary material for: Structures of the human spliceosomes before and after release of the ligated exon
Source: Cell Res. 2019 Feb 6;29(4):274–85. doi: 10.1038/s41422-019-0143-x (PMC6461851; doi:10.1038/s41422-019-0143-x)
Supplement: Supplementary file 5 — Supplementary Figure 5 [file 41422_2019_143_MOESM5_ESM.pdf]

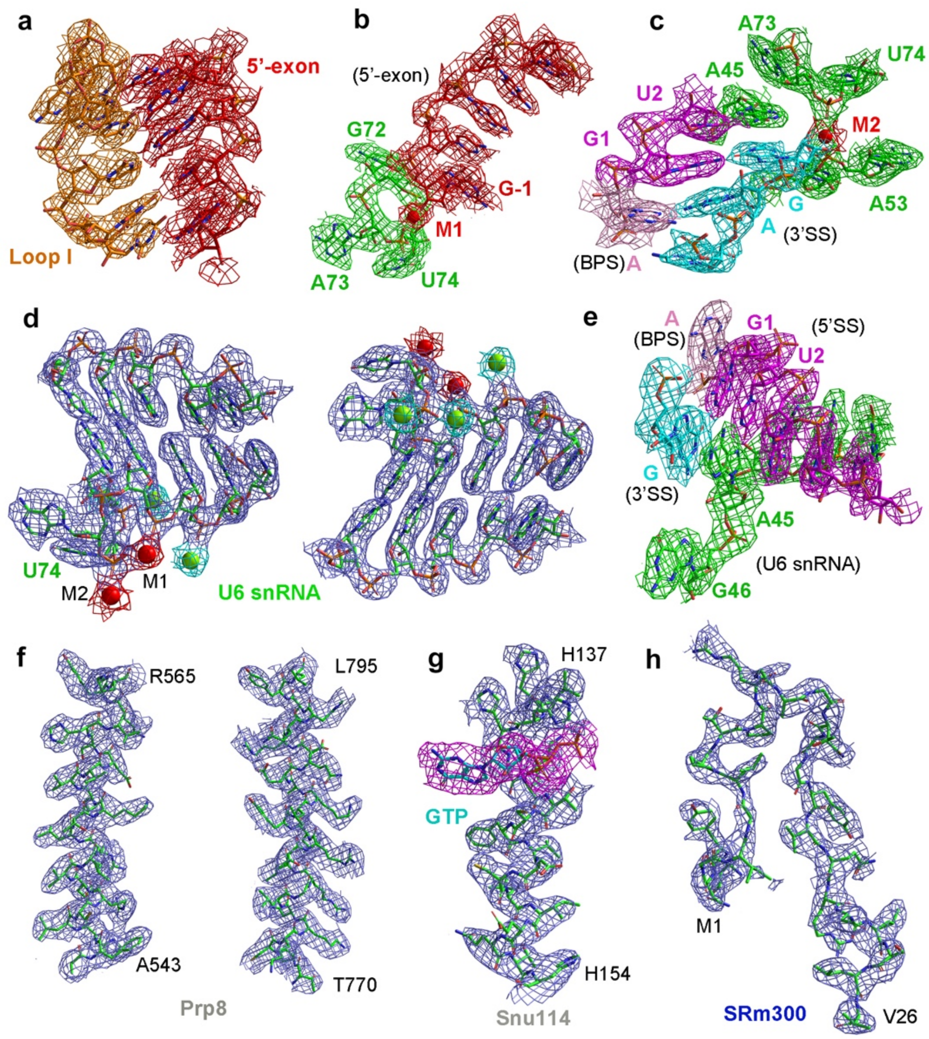

Supplementary information Figure S5. Representative EM density maps for select elements of the RNA and protein components in the human P complex. a, The EM density map for the duplex between 5'-exon and loop I of U5 snRNA. **b,** A close-up view on the EM density map surrounding the catalytic metal M1. **c,** A close-up view on the EM density map surrounding the 3'-splice site (3'SS). **d,** The EM density map for U6 snRNA, two catalytic metals (M1 and M2, colored red), and four structural metals (colored green). **e,** The EM density map of the duplex between 5'-splice site (5'SS) and U6 snRNA. **f,** Representative EM density maps of two helices from Prp8. **g,** A close-up view on the EM density map of GTP and a neighboring helix from Snu114. **h,** The EM density map of the splicing factor SRm300.
