# Supplementary material for: Structures of the human spliceosomes before and after release of the ligated exon
Source: Cell Res. 2019 Feb 6;29(4):274–85. doi: 10.1038/s41422-019-0143-x (PMC6461851; doi:10.1038/s41422-019-0143-x)
Supplement: Supplementary file 6 — Supplementary Figure 6 [file 41422_2019_143_MOESM6_ESM.pdf]

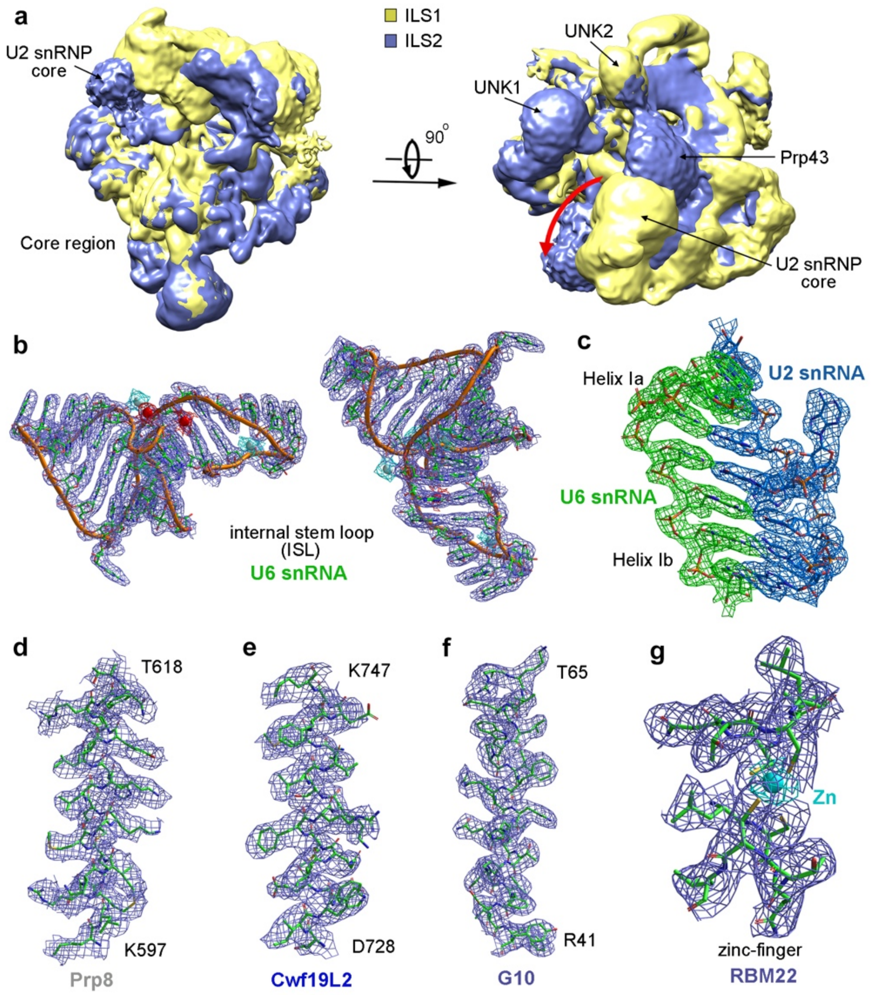

Supplementary information Figure S6. The EM density maps of the human ILS1 and ILS2 complexes. a, The overall EM density maps of the ILS1 and ILS2 complexes are low-pass filtered to 10 Å and overlaid together using Chimera. Two perpendicular views are shown. The core regions of the two ILS complexes are identical to each other. Compared to the ILS1 complex, the ILS2 complex has an extra lobe of density that is assigned to the ATPase/helicase Prp43. The U2 snRNP core has shifted during the ILS1-to-ILS2 transition. Each of the two ILS complexes contains two identical lumps of EM density near Prp43 that remain to be assigned. **b,** Two views of the EM density map for the internal stem loop (ISL) of U6 snRNA. **c,** A close-up view on the EM density map of helix I of the U2/U6 duplex. **d,** Representative EM density of one helix from Prp8. **e,** Representative EM density of one helix from Cwf19L2. **f,** Representative EM density of one helix from G10. **g,** A close-up view on the EM density of a zinc-finger from RBM22.
