# Supplementary material for: Structures of the human spliceosomes before and after release of the ligated exon
Source: Cell Res. 2019 Feb 6;29(4):274–85. doi: 10.1038/s41422-019-0143-x (PMC6461851; doi:10.1038/s41422-019-0143-x)
Supplement: Supplementary file 7 — Supplementary Figure 7 [file 41422_2019_143_MOESM7_ESM.pdf]

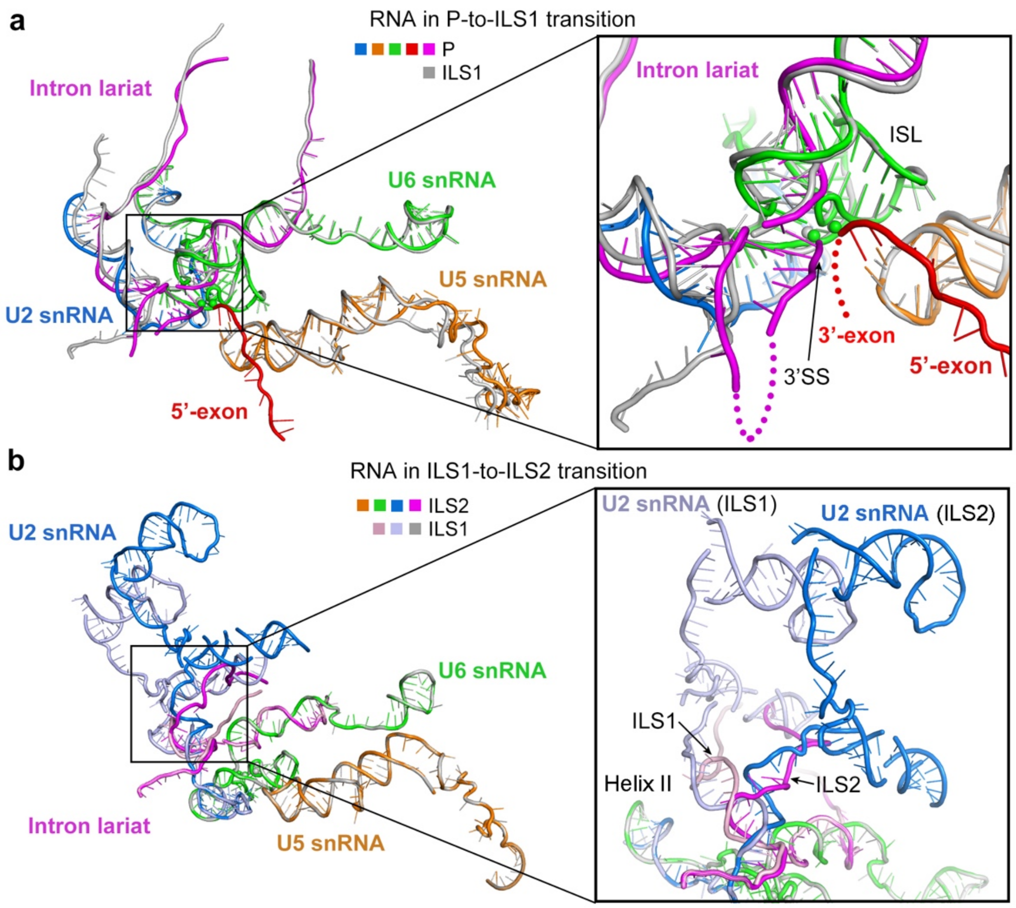

Supplementary information Figure S7. Comparison of the RNA elements and the splicing catalytic enter among the spliceosomal P, ILS1, and ILS2 complex. a, The RNA elements remain generally unchanged during the P-to-ILS1 transition, except that the ligated exon in the P complex has been dissociated in the ILS1 complex and the 3'-tail of the intron has rotated about 60 degrees away from the catalytic center. Shown here is the overlay of the overall RNA elements from the P and ILS1 complex (left panel) and a close-up view of the catalytic center (right panel). **b,** Rearrangements of the RNA elements during the ILS1-to-ILS2 transition. U5 and U6 snRNAs remain unchanged, whereas U2 snRNA is rotated about 40 degrees in the clockwise direction as shown. The U2/BPS duplex is also translocated.
