# Supplementary material for: Structures of the human spliceosomes before and after release of the ligated exon
Source: Cell Res. 2019 Feb 6;29(4):274–85. doi: 10.1038/s41422-019-0143-x (PMC6461851; doi:10.1038/s41422-019-0143-x)
Supplement: Supplementary file 8 — Supplementary Figure 8 [file 41422_2019_143_MOESM8_ESM.pdf]

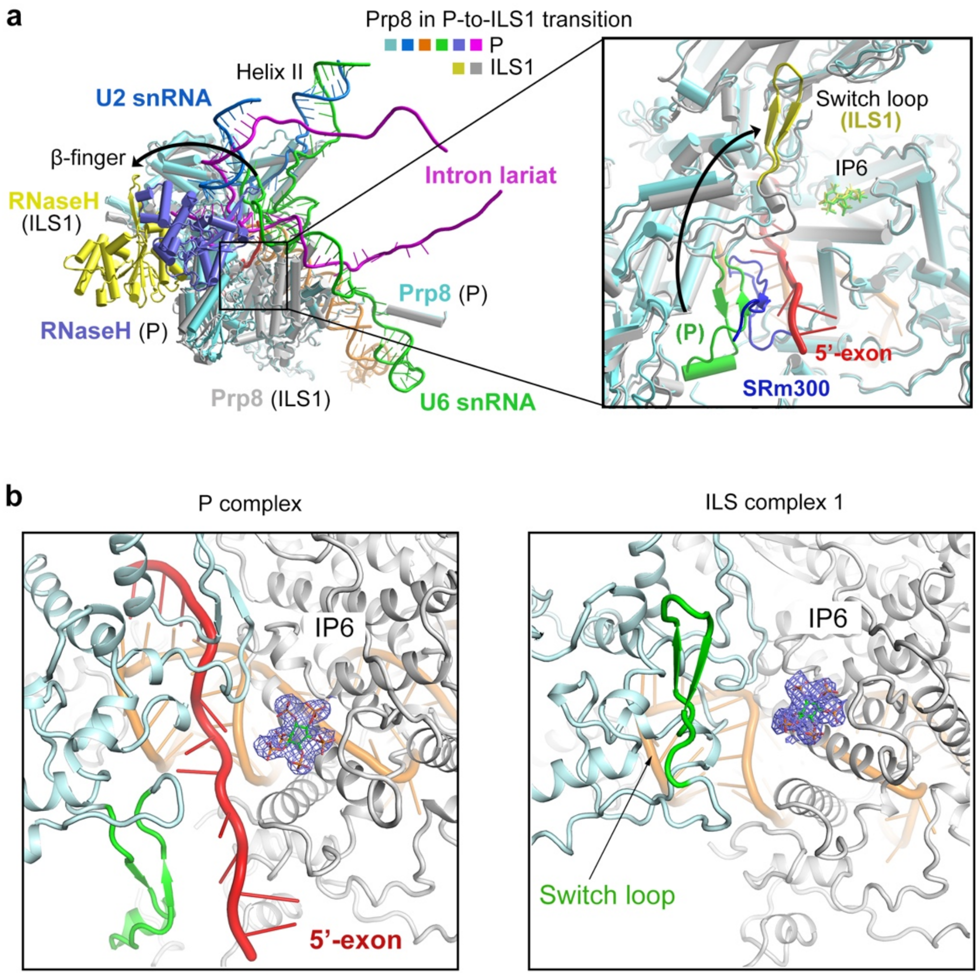

Supplementary information Figure S8. Conformational changes of Prp8 during the P-to-ILS1 transition. a, Superposition of Prp8 from the P and ILS1 complexes. The RNaseH-like domain from the P and ILS1 complex are colored blue and yellow, respectively. The N-domain and core from the P and ILS1 complexes are colored cyan and grey, respectively. The ß-finger is highlighted. In the right panel, a close-up view is shown on the Switch loop and a small molecule that has been tentatively assigned as inositol 1,2,3,4,5,6-hexaphosphate (IP6). **b,** Close-up views of IP6 from the P complex (left panel) and the ILS1 complex (right panel). The cryo-EM density maps for IP6 are shown.
