# Supplementary material for: Structures of the human spliceosomes before and after release of the ligated exon
Source: Cell Res. 2019 Feb 6;29(4):274–85. doi: 10.1038/s41422-019-0143-x (PMC6461851; doi:10.1038/s41422-019-0143-x)
Supplement: Supplementary file 9 — Supplementary Figure 9 [file 41422_2019_143_MOESM9_ESM.pdf]

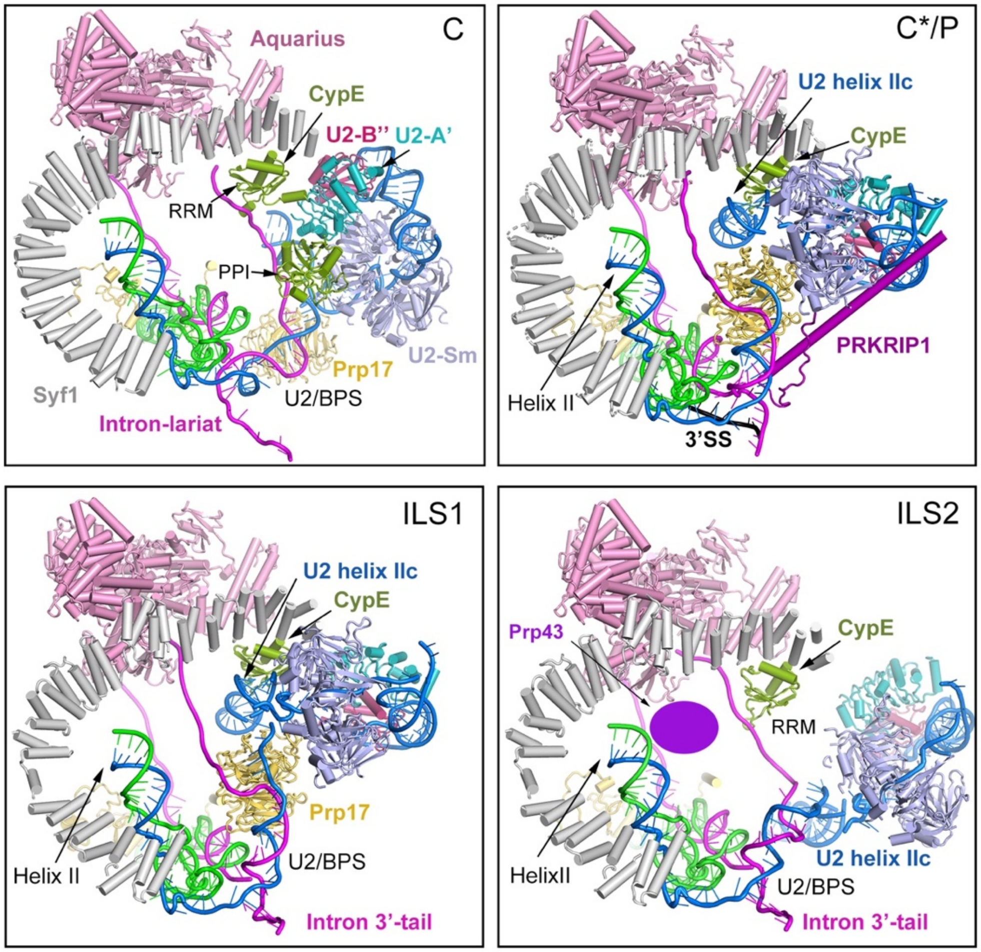

Supplementary information Figure S9. Movement of the U2 snRNP region during human spliceosome transition. U2 snRNP undergoes drastic translocation during remodeling of the human spliceosome. Shown here is the local conformations of the human spliceosomal C, C*/P, ILS1, and ILS2 complexes. In the C complex, the sequences upstream of the BPS are recognized by the RRM domain of CypE. Prp17 is yet to be loaded into the active site. During the C-to-C* transition, U2 Sm ring is translocated by about 100 Å to the N-terminal region of Syf1, and the U2/BPS duplex is rotated away from active site. The step II factor Prp17 is now loaded between the U2/BPS duplex and the ISL. The intron sequences move away from CypE, which interacts with U2 helix IIc in the C* complex. The splicing factor PRKRIP1 stabilizes the local conformation. In the P complex, the location of U2 snRNP remains unchanged. During the P-to-ILS1 transition, the local conformation remains unchanged except that PRKRIP1 is dissociated. In ILS2, Prp43 is recruited and located on the concave side of Syf1. The U2 snRNP core is translocated away from CypE, which is re-bound by the intron sequences upstream of the U2/BPS duplex. The WD40 domain of Prp17 is moved away from the active site and becomes disordered in ILS2 complex.
