# Supplementary material for: Structures of the human spliceosomes before and after release of the ligated exon
Source: Cell Res. 2019 Feb 6;29(4):274–85. doi: 10.1038/s41422-019-0143-x (PMC6461851; doi:10.1038/s41422-019-0143-x)
Supplement: Supplementary file 10 — Supplementary Table 1 [file 41422_2019_143_MOESM10_ESM.pdf]

Supplementary information, Table S1. Cryo-EM data collection and refinement statistics.

**Data collection**	
EM equipment	FEI Titan Krios
Voltage (kV)	300
Detector	Gatan K2
Pixel size (Å)	1.338
Electron dose (e-/Å^2)	45.6
Defocus range (μm)	0.8~1.8
**Reconstruction (P/ILS1/ILS2)**	
Software	RELION-2.0 & THUNDER
Number of used Particles	143,320/390,072/499,840
Accuracy of rotation (˚)	0.21/0.19/0.25
Accuracy of translation (Å)	0.23/0.18/0.25
Final Resolution (Å)	3.0/2.9/2.9
**Model building**	
Software	Coot
**Refinement (P/ILS1/ILS2)**	
Software	Refmac5.8
Map sharpening B-factor (Å^2)	-102.5/-93.3/-103.1
Average Fourier shell correlation	0.860/0.874/0.880
R-factor	0.31/0.30/0.30
**Model composition (P/ILS1/ILS2)**	
Protein residues	14184/10517/10900
RNA nucleotides	462/417/420
GTP	1/1/1
**Validation (P/ILS1/ILS2)**	
R.m.s deviations	
Bonds length (Å)	0.014/0.014/0.014
Bonds Angle (˚)	1.612/1.599/1.591
Ramachandran plot statistics (%)	
Preferred	89.50/84.31/84.22
Allowed	6.28/9.19/9.40
Outlier	4.22/6.50/6.39
Molprobity score	3.73/4.13/4.13
