# Supplementary material for: Structures of the human spliceosomes before and after release of the ligated exon
Source: Cell Res. 2019 Feb 6;29(4):274–85. doi: 10.1038/s41422-019-0143-x (PMC6461851; doi:10.1038/s41422-019-0143-x)
Supplement: Supplementary file 11 — Supplementary Table 2 [file 41422_2019_143_MOESM11_ESM.pdf]

Supplementary information, Table S2. Summary of model building for the human spliceosomal P complex.

	Molecule<br>Human/*S.pombe*/*S.cere*	Length	Domain/Region	PDB code	Modeling	Resolution (Å)	Chain ID
**U5 snRNP**	U5 snRNA	117	3:116	5XJC	HM	2.8~4.0	B
	Prp8/Spp42/Prp8	2335	56:662/675:2026/<br>Jab1/MPN (2067:2335)		HM<br>RD	2.8~4.0<br>8.0~10.0	A
	Snu114/Cwf10/Snu114	972	112:943		HM	2.8~4.0	C
	Brr2	2136	404:2125		RD	10.0~15.0	D
	U5-40K/Cwf17/-	357	WD40 domain		HM	3.5~4.5	E
	SmB,D1,D2,D3,E,F,G	-	Sm fold		RD	5.0~10.0	a-g
**U6 snRNP**	U6 snRNA	107 nt	1:97	5XJC	HM	2.8~4.0	F
**Pre-mRNA**	Pre-mRNA	-	84 nt	5XJC	HM	2.8~4.5	G
**U2 snRNP**	U2 RNA	188 nt	1:47<br>54:184	5XJC	HM<br>RD	3.0~4.5<br>6.0~10.0	H
	U2-A'/Lea1/Lea1	255	LRR domain	1A9N	RD	6.0~12.0	o
	U2-B"/Msl1/Msl1	225	RRM domain	1A9N	RD	6.0~12.0	p
	SmB,D1,D2,D3,E,F,G	-	Sm fold	4WZJ	RD	6.0~12.0	h-n
**Prp19/ NTC Complex**	Prp19	504	3:137	5XJC	RD	4.5~7.0	q-t
	Syf1/Cwf3/Syf1	855	1:800		RD	4.5~8.0	I
	Syf3/Cwf4/Clf1	848	179:215<br>TPR domain (216:446)<br>TPR domain (447:740)		HM<br>HM<br>RD	2.8~4.0<br>3.0~4.0<br>4.5~8.0	J
	Spf27/Cwf7/Snt309	225	14:222		HM	4.5~8.0	K
	Cdc5/Cdc5/Cef1	802	Myb Domain (6:108)<br>130:270<br>689:795		HM<br>HM<br>RD	2.8~3.0<br>3.0~4.0<br>4.5~8.0	L
	Syf2	201	113:243		HM	2.8~3.5	M
**NTC Related proteins**	G10/Cwf14/Bud31	144	1:144	5XJC	HM	3.0~4.0	N
	RBM22/Cwf5/Cwf2<br>/Ecm2/Cwc2	420	19:303		HM	3.0~3.5	O
	Ad-002/Cwf15/Cwc15	229	2:78/188:229		HM	2.8~3.5	P
	SKIP/Prp45/Prp45	536	51:316		HM	3.0~4.0	R
	PPIL1	166	Cyclophilin domain(8:166)		HM	3.0~3.5	S
	PRL1/Prp5/Prp46	514	WD40 domain (184:496)		HM	3.0~3.5	T
**IBC protein**	Aquarius/Cwf11/-	1485	19:1381	5XJC	RD	6.0~10.0	Q
**Known Splicing Factors**	SRm300/Cwf21/Cwc21	2296	1:26	5XJC	HM	2.8~3.5	U
	Cwc22/Cwf22/Cwc22	908	MIF4G domain (150:350)<br>MA3 domain (450:575)		HM<br>HM	4.0~8.0<br>3.0~4.0	V
	CypE/-/-	301	RRM domain (6:84)	3MDF	RD	8.0~10.0	y
	Prp17	579	83:273<br>WD40 domain (274:576)	5XJC	HM<br>HM	3.0~4.0<br>4.0~6.0	W
	PRKRIP1	184	51:142		HM	3.0~4.0	X
	Prp22	1220			RD	8.0~15.0	Y
	Slu7/	586	62:196/265:371		HM	3.0~4.5	Z
**EJC complex**	eIF4AIII/eIF4A/Fal1	411	22:407	2HYI	RD	8.0~15.0	u
	MAGOH/Mnh1/-	148	3:148		RD	8.0~15.0	v
	Y14/-/-	213	64:154		RD	8.0~15.0	w
	MLN51/-/-	741			RD	8.0~15.0	x

Under the column labeled "Molecule", proteins from human, *S. pombe*, and *S. cerevisiae* are colored black, red, and green, respectively. If the proteins from all three species have the same name, only a single name in black is indicated. Under the column labeled "Modeling", HM stands for homology modelling; RD stands for rigid docking and refinement.
