# Supplementary material for: Structures of the human spliceosomes before and after release of the ligated exon
Source: Cell Res. 2019 Feb 6;29(4):274–85. doi: 10.1038/s41422-019-0143-x (PMC6461851; doi:10.1038/s41422-019-0143-x)
Supplement: Supplementary file 12 — Supplementary Table 3 [file 41422_2019_143_MOESM12_ESM.pdf]

Supplementary information, Table S3. Summary of model building for the human spliceosomal ILS complex.

	Molecule Human/*S.pombe*/*S.cere*	Length	Domain/Region	PDB code	Modeling	Resolution (Å)	Chain ID
**U5 snRNP**	U5 snRNA	117	3:116	5XJC	HM	2.8~4.0	B
	Prp8/Spp42/Prp8	2335	56:662/675:2026		HM	2.8~4.0	A
	Snu114/Cwf10/Snu114	972	112:943		HM	2.8~4.0	C
	U5-40K/Cwf17/-	357	WD40 domain		HM	3.5~4.5	E
	SmB,D1,D2,D3,E,F,G	-	Sm fold		RD	4.0~8.0	a-g
**U6 snRNP**	U6 snRNA	107 nt	1:97	5XJC	HM	2.8~4.0	F
**Pre-mRNA**	Pre-mRNA	-	70 nt	5XJC	HM	2.8~4.5	G
**U2 snRNP**	U2 RNA	188 nt	1:47<br>54:184	5XJC	HM<br>RD	3.0~5.0<br>8~15.0	H
	U2-A'/Lea1/Lea1	255	LRR domain	1A9N	RD	8~15.0	o
	U2-B"/Msl1/Msl1	225	RRM domain	1A9N	RD	8~15.0	p
	SmB,D1,D2,D3,E,F,G	-	Sm fold	4WZJ	RD	8~15.0	h-n
**Prp19/ NTC Complex**	Prp19	504	3:137	5XJC	RD	4.5~7.0	q-t
	Syf1/Cwf3/Syf1	855	1:800		RD	4.5~8.0	I
	Syf3/Cwf4/Clf1	848	179:215<br>TPR domain (216:446)<br>TPR domain (447:740)		HM<br>HM<br>RD	2.8~4.0<br>3.0~4.0<br>4.5~8.0	J
	Spf27/Cwf7/Snt309	225	14:222		HM	4.5~8.0	K
	Cdc5/Cdc5/Cef1	802	Myb Domain (6:108)<br>130:270<br>689:795		HM<br>HM<br>RD	2.8~3.0<br>3.0~4.0<br>4.5~8.0	L
	Syf2	201	113:243		HM	2.8~3.5	M
**NTC Related proteins**	G10/Cwf14/Bud31	144	1:144	5XJC	HM	3.0~4.0	N
	RBM22/Cwf5/Cwf2 /Ecm2/Cwc2	420	19:303		HM	3.0~3.5	O
	Ad-002/Cwf15/Cwc15	229	2:78/188:229		HM	2.8~3.5	P
	SKIP/Prp45/Prp45	536	51:316		HM	3.0~4.0	R
	PPIL1	166	Cyclophilin domain(8:166)		HM	3.0~3.5	S
	PRL1/Prp5/Prp46	514	WD40 domain (184:496)		HM	3.0~3.5	T
**IBC protein**	Aquarius/Cwf11/-	1485	19:1381	5XJC	RD	6.0~10.0	Q
**Known Splicing Factors**	CypE/-/-	301	RRM domain (6:84)	3MDF	RD	8.0~10.0	y
	Prp17	579	83:273<br>(ILS1)WD40 domain (274:576)	5XJC	HM<br>HM	3.0~4.0<br>4.0~5.0	W
	Cwf19L2/	894	536:894	3JB9	HM	3.0~4.0	U
	Prp43(ILS2)	795		5XDR	RD	15.0~20.0	V

Under the column labeled "Molecule", proteins from human, *S. pombe*, and *S. cerevisiae* are colored black, red, and green, respectively. If the proteins from all three species have the same name, only a single name in black is indicated. Under the column labeled "Modeling", HM stands for homology modelling; RD stands for rigid docking and refinement.
