# Supplementary material for: Structures of the human spliceosomes before and after release of the ligated exon
Source: Cell Res. 2019 Feb 6;29(4):274–85. doi: 10.1038/s41422-019-0143-x (PMC6461851; doi:10.1038/s41422-019-0143-x)
Supplement: Supplementary file 13 — Supplementary Table 4 [file 41422_2019_143_MOESM13_ESM.pdf]

Supplementary information, Table S4. Summary of proteins and RNA components of human and yeast ILS complexes.

	Human (pdb: 6ID0 and 6ID1)	*S. pombe* (pdb: 3JB9)	*S. cerevisiae* (pdb:5Y88)
**U5 snRNP**	U5 snRNA	U5 snRNA	U5 snRNA
	Prp8	Spp42	Prp8
	Snu114	Cwf10	Snu114
	U5-40K	Cwf17	
	U5 Sm ring	U5 Sm ring	U5 Sm ring
**U6 snRNP**	U6 snRNA	U6 snRNA	U6 snRNA
**pre-mRNA**	intron lariat	intron lariat	intron lariat
**U2 snRNP**	U2 snRNA	U2 snRNA	U2 snRNA
	U2-A'	Lea1	Lea1
	U2-B"	Msl1	Msl1
	U2 Sm ring	U2 Sm ring	U2 Sm ring
**Prp19/NTC Complex**	Prp19	Cwf8	Prp19
	Syf1	Cwf3	Syf1
	Syf3	Cwf4	Clf1
	Spf27	Cwf7	Snt309
	Cdc5	cdc5	Cef1
	Syf2		Syf2
**NTC Related proteins**	G10	Cwf14	Bud31
	RBM22	Cwf5/Cwf2	Ecm2/Cwc2
	Ad-002	Cwf15	Cwc15
	SKIP	Prp45	Prp45
	PPIL1		
	PRL1	Prp5	Prp46
		Cwf2	
**IBC protein**	Aquarius	Cwf11	
**Splicing factors**	CypE		
	Prp17	Prp17	Prp17
	Cwf19L2	Cwf19	
	Prp43 (ILS2)		Prp43
			Ntr1
			Ntr2
			Cwc23
		Cyp1	

Protein and RNA elements of ILS complexes from human, *S. pombe*, and *S. cerevisiae* are summarized. Cells are left blank if there's no counterpart protein.
