# Supplementary material for: Structures of the human spliceosomes before and after release of the ligated exon
Source: Cell Res. 2019 Feb 6;29(4):274–85. doi: 10.1038/s41422-019-0143-x (PMC6461851; doi:10.1038/s41422-019-0143-x)
Supplement: Supplementary file 16 — Supplementary Movie Legends [file 41422_2019_143_MOESM16_ESM.pdf]

Supplementary information Movie S1. Human spliceosomal P-to-ILS1 transition.

Supplementary information Movie S2. Human spliceosomal ILS1-to-ILS2 transition.
